# An analysis of artificial intelligence automation in digital music streaming platforms for improving consumer subscription responses: a review

**DOI:** 10.3389/frai.2024.1515716

**Published:** 2025-01-15

**Authors:** Nontokozo Mokoena, Ibidun Christiana Obagbuwa

**Affiliations:** ^1^Centre for Applied Data Science, University of Johannesburg, Johannesburg, South Africa; ^2^Faculty of Natural and Applied Sciences, Department of Computer Science and Information Technology, Sol Plaatje University, Kimberley, South Africa

**Keywords:** artificial inteligence-AI, automation, music streaming applications, digital music subscription, digital music streaming platforms

## Abstract

The rapid adoption and evolving nature of artificial intelligence (AI) is playing a significant role in shaping the music streaming industry. AI has become a key player in transforming the digital music streaming industry, particularly in enhancing user experiences and driving subscription growth. Through AI automation, platforms personalize music recommendations, optimize subscription offerings, and improve customer support services. This article reviews the role of AI in driving consumer subscription behaviors on digital music streaming platforms (DMSP), with a focus on recommendation algorithms, dynamic pricing models, marketing automation, and the future of AI in the music industry. Potential challenges related to privacy, ethics, and algorithmic biases are also discussed, showcasing how AI is revolutionizing the music streaming industry.

## 1 Introduction

The digital music streaming industry has transformed how consumers access and engage with music (Chung et al., 2022). Platforms such as Spotify, Apple Music, and Amazon Music have become dominant forces in the market, offering users access to vast music libraries at their fingertips (Guo, 2023). As the competition among these platforms intensifies, Artificial Intelligence (AI) has emerged as a critical tool for enhancing user experiences and driving consumer subscriptions. AI-driven automation is transforming the digital music ecosystem, enabling platforms to personalize music recommendations, optimize subscription models, and improve customer service (Wang et al., 2023). These advancements not only streamline user interactions but also increase engagement and retention rates, which are vital for the success of subscription-based business models.

Fast tracking the era of music streaming services is the development and use of smartphones, mobile applications and smart televisions, which has revolutionized the music industry seeing consumers choosing access to music over ownership of music (Mouawad and Gavrilova, 2020). The music industry has experienced a revenue resurgence from music streaming after a decade of declining revenue (Future Europe Journal, 2024). This vast access to music catalogs has contributed to decreasing music piracy, reviving the music industry (Hesmondhalgh, 2021) and boosting revenue. Music streaming platforms are miniature copy of digital transformation and present a good catalyst of how distributive technologies can shift industries, develop new value chains and strategies to connect with audiences in unprecedent ways.

AI’s role in digital music streaming goes beyond simple automation of repetitive tasks. It enables platforms to analyze vast amounts of user data to predict behavior, personalize content, audience targeting, segmentation and even forecasting trends (Gkikas and Theodoridis, 2022). From advanced recommendation algorithms to dynamic pricing strategies and automated marketing campaigns, AI is becoming increasingly influential in shaping user experiences and subscription behaviors. As users encounter more personalized and seamless interactions, they are more likely to engage deeply with the platform, ultimately leading to experiential brand loyalty (Obiegbu and Larsen, 2024), higher subscription conversion and retention rates.

However, the widespread use of AI in music streaming platforms also presents significant challenges, forcing these technologies to consider concerns about their ethicality (Bonjack and Trujillo, 2024). The use of personal data to enhance the user experience raises concerns around privacy and data security. Furthermore, there are growing concerns about algorithmic biases that may limit the diversity of music that users are exposed to, potentially narrowing their musical horizons. As AI continues to evolve, it is essential to understand how these technologies influence user behavior and consider the ethical implications of their deployment.

This review aims to explore how AI automation influences consumer subscription responses in digital music streaming platforms. By examining the key areas where AI is deployed such as recommendation systems, subscription pricing, marketing, and customer support we can gain insights into how these platforms leverage AI to enhance user engagement. Additionally, this review will discuss the challenges associated with AI in the streaming industry, including data privacy concerns and the potential for algorithmic bias, and how platforms can address these issues to foster sustainable growth.

## 2 AI-driven personalization and consumer engagement

### 2.1 AI-powered recommendation systems

One of the most impactful uses of AI in music streaming platforms is the recommendation system. AI algorithms analyze user data, such as listening habits, likes, skips, and search queries, to predict and recommend new music to users. This system, driven by machine learning techniques like collaborative filtering and content-based filtering, has improved user engagement and satisfaction.

AI recommendation systems have become a crucial component in the digital landscape influencing and shaping user behavior. Carole et al. (2024) found that 80% of consumers are more likely to purchase a service or product from a brand that provides personalized experiences. Spotify’s “Discover Weekly” and Apple Music’s “For You” are prime examples of how AI generates personalized playlists by identifying patterns in user behavior. Research has shown that personalized recommendations increase user loyalty and are a primary factor influencing the decision to subscribe to premium services.

Hallikainen et al. (2019) mention that the value of AI comes from the consumers perception and acceptance of the technology, the level of privacy provided, trust and overall experience generated. Consumers have been seen to be spending extended time shopping online with multiple choices at their fingertips, AI recommendation systems help increase efficiency by recommending relevant products or services to consumers which reduces their spend time and leads them choosing services or online music streaming subscription packages that will suit their needs best (Habil et al., 2023). Recommendation systems enhance the consumer’s experience by providing recommendations that meet their selection criteria and in turn satisfy their wants and needs (Liao et al., 2021).

AI’s algorithms assist in collecting systematic data of the user’s behavior when engaging with the digital music streaming platform. These algorithms are used to understand the users’ preferences, likes and dislikes when it comes to their music, then matching it with similar artist catalogs, albums or songs which are then presented to the user (Niyazov et al., 2021). Music recommendation systems on DMSP play a critical role that can drive discovery of new music and artists presented to the user which aids in keeping them engaged and continuously coming back on the DMSP (Kadam, 2023). AI recommendation engines can be scalable, highly personalized and optimized to reach the desired outcome such as longer listening times (Bhardwaj et al., 2021), which is key in keeping users engaged as they discover new artists and music without them feeling disconnected or that their experience is stale or boring (Bello and Garcia, 2021). Implementing responsible AI practices has a become essential, especially with the rise of ethical and social concerns in the digital landscape. A study by Murindanyi et al. (2024) demonstrates the potential of responsible AI to produce personalized recommendations for users while decreasing possible risks. Highlighting how users can utilize AI based music recommendation systems that can offer personalized music recommendations while decreasing risk through enforcing accountability by transparency and explainability.

AI recommendation systems make content research efficient, easier, user centric and enriching (Sivasankari et al., 2024). Digital platforms such as online music streaming, social media, and e-learning utilize recommendation systems to solve the problem of information overloading (Ayemowa et al., 2024). Recommendation systems now play an important role in the daily lives of online users, and many authors are currently exploring different ways in which recommendation systems algorithms can improve customer stratification and the systems accuracy (Gao et al., 2021).

### 2.2 Contextual and hyper-personalization

In addition to analysing listening behavior, AI systems now account for contextual factors like time, location, and mood to deliver hyper-personalized recommendations. For example, AI might suggest workout music in the morning and calming tunes in the evening based on a user’s previous behavior. This level of personalization creates a unique user experience, fostering a stronger emotional connection between the consumer and the platform. In turn, this results in higher subscription rates and customer retention.

Personalization plays a vital role in the way companies differentiate themselves (Winter et al., 2021), and in delivering personalized content through recommendations systems and retargeted advertisements. Through companies collecting consumer data, they can better generate consumer profiling, personalized recommendations, new behavior insights and accurate targeting (Hayes et al., 2021). These new technologies have opened the doors for marketers to be able to tailor highly effective campaigns that are hyper personalized and relevant (Grigorios et al., 2022). Online behavioral targeting enables online music streaming platforms to offer personalized adverts (Puri and Mohan, 2020), recommendation systems help create value for both the consumer and the digital music streaming platform through interchangeable facets of contextual and hyper personalization (Habil et al., 2023).

AI powered hyper personalized music recommendation systems have the ability to influence the user’s emotional state. Sharma et al. (2024) mention that through merging problem solving and emotional wellbeing “Feel Good AI, a voice enabled emotion-based music recommendation system” technology can greatly enhance a user’s quality of life, more especially with the rise of mental wellness. Benefits that come with implementing AI-powered recommendation systems are the ability to personalize engagements and content delivery, meeting customers’ expectations, boosting and optimising the DMSP performance and reducing frustration through providing a seamless user experience, however privacy concerns are still an issue where DMSPs need to establish trust with consumers (Carole et al., 2024).

Personalized platforms such as digital music streaming strive to create and deliver personalized content to users based on their user profile and understanding individual user behavior, it is however important to deliver satisfying content in specific and differentiated context (Kim et al., 2023). This strategy can aid in improving user engagement on the platform and in return generate increased revenue for the DMSP.

Ethical considerations play an important role in the delivery of hyper personalization (Moyeenudin et al., 2021; Vemuganti, 2022). Contextual and (Rane et al., 2023) hyper personalization enables companies to forge emotional connections with users by displaying their understanding of users’ needs and wants on the platform, which transcends the transactional encounter and creates consumer trust and consumer brand advocates. Unlike conventional personalization, hyper personalization dives deeper into exploring details such as user behavior, historical interaction and individual preferences. This enhances user engagement which increases retention especially for subscription-based organizations where consumers find ongoing value through customized offerings, which create a sense of exclusivity.

Hyper personalization enables organizations to identify user pain points with the aim to reduce any frustrations or friction, to ultimately improve customer satisfaction through providing seamless, efficient and effective user experience operations (Bashar et al., 2022). However contextual and hyper personalization relies highly on correct and accurate data, it’s crucial to implement data quality assurance procedures and integration across different systems and departments because inaccurate data can lead to misguided marketing communication and personalization efforts (Rane et al., 2023), which can harm user relationships and have a negative impact on the DMSP brand. Having an ethical balance between customization and privacy is important to create consumer trust and avoid misusing consumer data (Rane and Jayaraj, 2022; Rane, 2023).

## 3 AI-optimized subscription models

### 3.1 Dynamic pricing models

AI automation has also enabled dynamic pricing models, which allow music streaming platforms to offer personalized subscription options. By analysing user demographics, income levels, and consumption habits, AI can determine the optimal subscription price points and create customized promotions. This approach increases the chances of converting free-tier users into paying subscribers by offering subscription tiers or limited-time offers that appeal to individual user needs.

Service companies need to be careful of the dynamic pricing approach as many consumers see it as unfair and it creates price confusion which can lead to negative word of mouth for a company or brand (Bambauer-Sachse and Young, 2024). Service companies use dynamic pricing models where sellers’ base prices on what individual consumers are willing to pay. Dynamic prices vary continuously based on algorithms, engaging price advantaged consumers who tend to react positively to dynamic pricing especially if it’s positioned as a discount (Keller et al., 2022). While price dis-advantaged consumers tend to react negatively, especially with the rise of social media consumers comparing how much they paid for a similar product or service from the same company, thus creating a sense of price discrimination, unfairness and confusion (Schmidt et al., 2020; Xue et al., 2020). According to Bambauer-Sachse and Young (2024) dynamic pricing usage should be limited or avoided especially when there is a high risk of reputational damage and negative word of mouth among price dis-advantaged consumers.

Big data analytics plays a key role in enabling organizations to adjust subscriptions fees and content pricing, through analysing consumer behaviors, historical content and market trends, empowering organizations to make data driven decisions (Ahmed and Abdulkareem, 2023). Through analysing consumer patterns on platforms, DMSPs can identify consumer segments who are willing to pay more for additional or exclusive content, premium features and new music releases before they are available to others. Through introducing different tiered pricing packages, they can offer different subscription levels, where each tier has certain benefits, features, and pricing structures. This approach allows DMSPs to target different consumer segments with additional revenue from those willing to pay more for enhanced customer experiences (Athmaja et al., 2017).

Organizations like Uber, Airbnb and Amazon have successfully been utilising AI for dynamic pricing effectively, adapting prices based on consumer behaviour, real time supply and demand factors, and competitive pricing (Gazi et al., 2024). With the integration of AI, dynamic pricing has becoming highly efficient, enabling organizations to analyze and interpret vast data in real time, allowing them to instantaneously adjust their pricing in efforts to remain competitive (Awais, 2024). For instance, Uber’s pricing mechanisms leverages AI to adjust trip fares according to the demand levels in a particular area, ensuring that the company can maximize revenue and can match the supply with demand. Dynamic pricing can lead to short term revenue, however if not managed effectively it can lead to customer churn long term (McMurtrey and Kasowaki, 2023), to address this issue AI systems can incorporate personalized loyalty programs into their pricing strategies, managing short term revenue optimization with customer retention (Awais, 2024).

Dynamic prices enable organizations to explore different offerings presented to consumers, allowing them to investigate what works and does not work in certain demographics or geographical areas. Some DMSPs offer premium subscription prices for exclusive content and releases, they also explore promotional pricing or limited time discounts to draw attention to potential new consumers and possibly convert them into monthly subscribers. By understanding the consumers’ willingness to pay, perception, preferences and value, DMSPs can learn how to strike an ethical and good balance of dynamic pricing strategies. Organizations can build consumer trust and loyalty through offering personalized pricing options and benefits (Ahmed and Abdulkareem, 2023).

### 3.2 Predictive analytics for retention

AI also plays a significant role in reducing churn rates by predicting when users are likely to cancel their subscriptions. Platforms use AI-driven predictive analytics to monitor user behavior and identify patterns associated with declining engagement, such as decreased listening time or frequent skips. When AI flags these patterns, platforms can respond by offering retention incentives like discounts or personalized offers, thereby preventing subscription cancelations.

It is easier to keep existing users than to acquire new ones, therefore it is important for digital music streaming platforms to identify churning signals earlier, in efforts to offer the user incentives to make them stay (Gaddam and Kadali, 2022) such as offering an exclusive preview of a certain feature or content. Predictive analyses play a vital role in digital businesses, enabling them to collect large datasets of user behavior and preferences. Through offering personalized recommendations using predictive analytics can assist DMSPs to capture the user’s attention and keep them engaged longer on their platform. By analysing their users’ behavior, listening habits, patterns, and preferences, Spotify uses data analytics to capture the user’s attention through offering relevant personalized content (Jawad and Al-Bakry, 2023).

Digital music streaming platforms using predictive analytics for retention enables the continuous improvement of its recommendation systems and the overall enhanced customer experience (Seifert et al., 2023). It’s crucial for digital businesses to leverage data analytics collected to employ effective and relevant user recommendations algorithms which enhance the users brand experience, increases brand loyalty and the retention rate (Philip and Pradiani, 2024). Data analytics used correctly will enable DMSPs to have a broader view of their users, enabling them to customize individualized algorithms to suit the users’ preferences, which increases the likelihood of the user feeling understood, and increases their listening time and the probability of them sharing content or playlists to other digital platforms for their network to see and engage with.

The use of this AI predictive analysis tool can contribute to improved user retention were the analysis of user behavior and patterns enable DMSPs to identify at risk users and deploy targeted mechanisms and strategies such as contextual and hyper personalization recommendations to decrease churn and increase customer loyalty (Zhao et al., 2023). By leveraging predictive data analytics DMSPs can effectively target consumers based on their likes and preferences and provide enhanced advertising effectiveness based on the user demographics and interests resulting in increased click through rates and advertisement revenue (Tarigan et al., 2023). Strategically leveraging data analytics provides monetisation opportunities through targeted initiatives, advertising and partnerships with artists and brands which can drive profitability and growth.

## 4 Targeted marketing and consumer acquisition

### 4.1 AI in marketing automation

AI has transformed the marketing landscape for digital music streaming platforms by automating targeted marketing campaigns. Platforms use AI to segment their audiences based on behavior, preferences, and demographics, allowing for more effective advertising. Tailored promotions and ads can be delivered to specific user groups, increasing the likelihood of converting free-tier users into paid subscribers.

AI-powered marketing extends beyond email campaigns and advertisements. AI can also use social media activity to gauge trends and predict emerging music tastes within specific communities, helping platforms stay ahead of consumer demands. Leveraging the use of AI data analytics enables organizations to implement personalized marketing strategies and deliver customized content offerings to consumers (Kedi et al., 2024). This drives engagement and loyalty and enhances a positive customer experience which is an important factor in today’s competitive digital landscape (Adewumi et al., 2024; Aldoseri et al., 2024). Automation is one of the most trendy and crucial marketing tools with its ability to automate repetitive tasks, improve efficiency and influence strategic aspects such as targeting, programming and segmentation (Hoffman et al., 2022).

AI algorithms are central to how music streaming platforms operate. They have significantly influenced content curation, user experience and music discovery on digital music streaming platforms. AI algorithmic systems have also been accused of perpetuating biases with its ability to potentially positively influence a user’s choice by inserting new artists in the forefront (Henry et al., 2024). Widely adopted AI algorithms on music streaming platforms such collaborative filtering powered by deep learning assist with providing suggestions to users based only on their likes and dislikes, influencing the type of playlists, genres and artists recommended by the DMSP (Torkashvand et al., 2023). Deep learning techniques play a vibrant role in recommendation systems through enhancing content relevance and improving user satisfaction by analysing feedback in real time and adjusting to evolving user preferences (Durani, 2023). These algorithms facilitate positive user experience through examining and understanding user behavior and interactions on the DMSP.

AI marketing automation does not only reduce costs and provide relief to mundane tasks, but is central to knowledge management (Jarrahi et al., 2023), providing data organization and structure. Marketing automation improves customer satisfaction through its ability to provide specific information communication to users which influences the user’s perception of the organization (Rajpurohit et al., 2023). Strategic uses of AI marketing automation can increase the user’s attachment to the specific DMSP, especially if they give opportunities to customers to voice their queries and comments which can serve as feedback data to help DMSPs improve their services and provide good customer service (Alomari et al., 2020). AI powered marketing automation enables DMSPs to streamline their marketing communication efforts to users with greater efficiency and accuracy. It’s important to note that effective marketing is influenced by data driven insights and decisions, where DMSPs leverage on AI algorithms to identify opportunities and obtain a deeper understanding of its users to better satisfy their needs and wants. AI marketing automation is a tool, and not a strategy (Sheth, 2020), therefore it is imperative for DMSPs to understand their audience, goals and resources to effectively leverage AI in marketing automation (Kaperonis, 2024).

Using AI automation to respond promptly to customers can improve customer service and communication. According to Santiago (2023) 62% of consumers prefer to communicate with a customer service AI agent than wait several minutes to engage with a human agent. Especially for situations that are surface level and do not require complex problem solving. AI agents or bots respond instantly to customers, saving time and costs. By constantly providing around the clock support 24/7, AI agents significantly enhance customer experience (Kaperonis, 2024). Leveraging AI in marketing automation involves identifying the correct software’s, tools and platforms to enable DMSPs to reach their marketing goals. The implementation of AI in marketing automation is exciting with excellent opportunities, however it is not devoid of challenges such as data privacy concerns and various ethical considerations (Martin, 2019; Zhou and Piramuthu, 2013).

### 4.2 Social media integration

AI also helps integrate music streaming platforms with social media, allowing users to discover new music through social connections. By analysing social interactions, AI can recommend playlists based on friends’ listening activities, trending music, and even songs shared within certain networks. This social aspect of AI integration is particularly effective among younger consumers, who are more likely to engage with music through social media platforms like Instagram and TikTok.

Modern musicians use social media as one of their main drivers to share music with audiences and connect with fans on platforms like Instagram, Facebook, X and Tik Tok (Nwagwu and Akintoye, 2023). Most of these social media platforms provide artists or users with a business page that gives them access to their specific data analytics to inform their content delivery, relevance and overall engagement which can inform short- or long-term strategy. AI driven data analytics on social media provides insights that highlight audience demographics, watch time, and targeted advertising features. Some argue that social media is the new streaming and is not just for music marketing anymore, but it is its own form of music consumption, with the increasing availability of tools that enable users to play with lyrics, remixes and user generated content stitches (Kahlert, 2024).

AI is a key competitive advantage factor that a lot of social media platforms are early adopters of (Zhang et al., 2024). Through creativity and innovation digital music streaming platforms and social media platforms can continue to differentiate their service features and highlight their unique selling points to users in order to stay relevant in the highly competitive digital landscape. Furthermore, collaborations and brand partnerships with different online stakeholders for example Instagram and Deezer where they champion trending burning issues or address societal challenges or wins can continue to revive the brands perception among the user through creating online visibility across channels.

Through better content matching, music recommendations and social connections, value can be created for users on these platforms increasing their spend time and sharing content across platforms. Many media platforms are now trying to be the everything app and Tik Tok is currently the leader of the “everything app” where users can post pictures, create videos, insert music, stitch videos, host lives, have options to multiple effects features and more, meaning they moving to e-commerce, game publishing, music distribution, live streaming and subscriptions (Huang, 2023) making it the one stop app. However, a huge potential risk in this approach is platforms having an identity crisis and losing their unique selling point (Zhang et al., 2024).

Undeniably, social media has influenced and, in some cases, transformed how many online businesses sell their products and services, and implementation of AI enables the rapid forms of data collection and analytics from user behaviour on the platform (Yadav et al., 2024). AI in social media provides high value to businesses, improved audience analysis, enhanced user engagement and content optimisation (Saheb et al., 2024).

## 5 AI in customer support and experience

### 5.1 AI Chatbots and virtual assistants

AI-powered virtual assistants and chatbots have become common features in digital music streaming platforms, providing instant customer support without the need for human intervention. These AI systems can handle a variety of tasks, including helping users troubleshoot subscription issues, answering questions about plans, and offering personalized recommendations. Automated customer service improves user experience and satisfaction, which in turn helps retain subscribers.

According to Khan and Iqbal (2020) AI chatbots and virtual assistants have completely altered the world of customer service, especially in digital marketing benefiting both the user and service provider. One of the biggest benefits of AI chatbots is its 24/7 support availability, specifically in the current age of instant gratification, users prefer fast solutions to their problems whether it is fixing account issues or subscriptions problems. AI technologies have revolutionized the way users interact with digital platforms (Kaperonis, 2024). AI chatbots offer quick replies for easy questions, reducing user waiting time and freeing up complex questions for human agents.

Through data analytics, chatbots can offer personalized assistance (Volkmar et al., 2022), recommending new music or podcasts that are associated to the user’s listening habits. Fun interactions instigated by AI chatbots such as music quizzes or creating playlists aligned to the users listening history can increase user engagement, listening time and encourage them to come back frequently on the platform. It is forecasted that the chatbot market will reach an estimated 1.25 billion U.S dollars by year 2025, seeing an impressive increase from 2016 when the market was 190.8 million U.S dollars (Statista, 2022). Chatbot’s aid in managing subscriptions using AI automation to assist users with renewing subscriptions, upgrading to a premium plan and also canceling subscriptions. Chatbots and virtual assistants will become more advanced and integrated with other technologies, cross cutting to user’s social media allowing them to share songs or playlists, interconnected with voice assistance, smart home devices and ultimately enabling users to connect across multiple digital channels (Huseynov, 2023).

As technology continues to evolve, so will the role of chatbots and virtual assistants, currently DMSPs use chatbots to assist users to discover new music and manage accounts. In future, predictive analytics will be used by chatbots to generate hyper personalization to keep users engaged and satisfied (DeepConverse Blog, 2024). Digital music streaming platforms have truly transformed consumers listening habits and the use of chatbots advances its operations. Wang et al. (2022) suggests that both the routine and innovative utilization of chatbots has the possibility to boost business agility. Vijayakumar (2024) research found chatbots and virtual assistants to enhance innovation that drives positive customer service practices, increase customer loyalty and increase positive user experience. More companies’ customer interfaces are being transformed by these technologies from being people oriented to being technology orientated (Konya-Baumbach et al., 2023). Huseynov (2023) mentions that as more companies implement the aid of chatbots and virtual assistants, ethical considerations should also be in the forefront of their processes, ensuring customers are aware who they are engaging to increase the levels of trust and comfortability and avoid confusion.

### 5.2 Sentiment analysis and feedback loops

Using natural language processing (NLP), AI can analyze user feedback, reviews, and social media posts to gauge overall customer sentiment. By detecting dissatisfaction or negative feedback early, platforms can proactively respond with solutions, offers, or improvements. These feedback loops, powered by AI, help platforms maintain a positive relationship with consumers and improve their service offerings to retain subscribers.

By leveraging user sentiment, organizations gain valuable information that informs their decision making, enabling the effective rollout of impactful marketing strategies, ultimately leading to an improved customer experience (Bharadwaj, 2023). Businesses get to satisfy their consumer needs by adapting their offerings which leads to customer loyalty by understanding and analysing sentiments. Consumers like sharing their opinion online about various topics, products and services, and organizations have found a way to capitalize on user opinions to further understand consumer feelings toward their brands, whether positive, negative or neutral (Çano, 2018).

Sentiment analysis is widely used by digital music streaming platforms as a key factor for recommendation systems to generate advertisements, through analysing user opinions DMSPs can measure and adjust marketing campaigns (Khan et al., 2024). App reviews serve as valuable feedback loops for organizations, with various attitudes from user comments displaying different program attributes, features and themes. Analysing these perspectives in depth assists software developers to understand users’ experience on their platform and factors that directly impact app sales and downloads (Liang et al., 2015; Nicolai et al., 2019).

Privacy concerns loom around sentimental analysis especially when organizations analyze user opinions on media platforms such as social media without their knowledge or consent which can cause privacy risks (Lee et al., 2022). Federated Learning is a privacy preserving technique which enables AI model training to utilize decentralized user data, without sharing raw data, Federated Learning is still in its early stages and being explored by researchers (Bansal and Kumar, 2022; Nagy et al., 2023; Sakhare and Shaik, 2023).

Sentimental analysis has proven to be significant in understanding market trends, public opinion and consumer feedback, future research should work on sentimental analysis ethical framework to reduce the risk of data exposure (Rahman Jim et al., 2024).

## 6 Challenges and ethical considerations

### 6.1 Data privacy

Despite the many benefits of AI automation, the use of personal data to drive recommendations and marketing campaigns raises concerns about privacy. Music streaming platforms collect vast amounts of user data, including listening habits, location, and social activity, which may lead to potential data misuse or breaches. Transparent data collection policies and stronger security measures are necessary to protect consumer privacy.

Central to AI advancements is the need to implement relevant frameworks and policies to guard the use of collected user data in the digital landscape (Yanamala and Suryadevara, 2023). Music industry players are calling for more increased accountability, fairness and transparent practices from music streaming platforms around their use of data and the distribution of streaming royalties to stakeholders. According to Vilmakumar et al. (2021), privacy concerns are vital to be considered as they greatly influence consumer trust.

Innovations such as Federated Learning mitigates data privacy risks by using AI model training associated with centralized data repositories, enabling the use of sensitive domains. There’s a crucial and urgent need to implement concerted efforts into improving security in privacy and data protection AI systems (Sebastian, 2023). Differential privacy offers another form of enhanced AI applications data privacy where individuals’ privacy is protected by providing a mathematical framework of statistical analyses implemented on sensitive datasets (Dwork, 2008), allowing for meaningful data analysis. Federated Learning and Differential Privacy are important technological innovations that represent the advancements in data privacy issues without the compromise of AI automation performance. However, more research needs to be explored on these technologies focusing on their scalability and applicability across multiple sectors (Yanamala and Suryadevara, 2023).

Some privacy issues in user collected data range from the risk of user exploited data used to train AI models, which raises ethical issues. AI technologies are not always welcomed by all stakeholders, due to the amount of data collected and analyzed (Kronemann et al., 2023). Biased algorithms potentially discriminating against one group or applying prejudice because of skewed inputted data can reinforce stereotypes and create consumer mistrust. There’s a lack of transparency regarding the usage of user data, many users have no idea how their data is being leveraged behind the scenes. Surveillance and monitoring “the watching eye” effect (Hu and Min, 2023) raises serious ethical concerns especially when AI is conscripted and encrypted into monitoring users online behaviour and facial pattern recognition. It is therefore critical for music streaming platforms to get user consent when implementing these technologies in efforts to reduce risk (Samba, 2024).

As AI developments advance in this continually changing digital landscape and to make it safe, many regulatory frameworks exist to protect data privacy such as the Protection of Personal Information Act (POPI Act), which is legislation that governs the law of privacy and data protection in South Africa. The General Data Protection Regulation (GDPR) (Yanamala and Suryadevara, 2023), Europe’s landmark that instigated this new era of data transparency, privacy and user control, and the California Consumer Privacy Act (CCPA) (Sherbini, 2023) amongst others.

Music streaming platforms must comply with data privacy laws ensuring that they obtain the users consent to retrieve their personal data while on their DMSP and provide clear language in how their data will be utilized (Ademeje Solicitors, 2024). DSMPs should give users the ability to opt in or out of data collection, this includes marketing communications. By complying with data privacy laws, music streaming platforms can create user trust, enhance data protection and foster sustainable relationships with stakeholders. According to Tsaaro (2021) it is the responsibility of the DMSP and the user to prioritize data privacy and work together to create a safe and secure environment while enjoying the music offering. Balancing offering personalized services and user privacy is essential to managing trust and avoiding legal repercussions. For instance, Spotify enables users to control advertisement personalization settings to ensure it suits their privacy preferences, and allows users to download data reports (Stål, 2021). Spotify also incorporates privacy preserving practice that provide the user the option to filter out explicit content or exclude certain playlists from interrupting and influencing their taste profile (Spotify.com, 2021). Transparency has the ability to provide insights into fairness (Dinnissen and Bauer, 2023), by integrating transparency initiatives like user access to data, clear privacy policies, data use and personalization, music streaming services comply with GDPR data protection laws which ensures that users are fully aware and in control of their data sharing. According to Pu et al. (2011) user control positively impacts user trust and satisfaction. Consent management practices such as opt in features, cookie management systems and granular consent options used mostly by YouTube Music and Spotify (Stål, 2021) allows users to choose if they want their listening activity on the DMSP to contribute to personalized recommendations or if they prefer to enable or disable targeted advertising. Such privacy preserving methods and options play a significant role in building user trust on DMSPs.

Strategies to manage and mitigate data privacy range from data minimization, where AI does not collect everything, but only key necessary information for AI application and analysis. Privacy by design, minimises data breaches where a security system is built into the AI, guiding its behavior in collecting data ethically and integrating solutions such as a cloud contract management into its coding (Samba, 2024). AI industry associations should play a central role in establishing guidelines and frameworks that constitute as standard practice, i.e., network identity verification systems in efforts to enhance data privacy systems and continuously consider ethical practices (Huang, 2023). Implementation of data anonymisation (Sebastian, 2023) and pseudonymisation, enabling AI to utilize user data to train models however, protecting the user’s identity making it impossible to trace the user. Strong security measures such as the use of encryption and strict access control, transparency and explainability in how AI reaches its conclusion about our data are all strategic efforts that can be implemented to manage and mitigate data privacy concerns. It is of ethical importance to ensure data protection and privacy is implemented across all AI systems (Khoury, 2023).

### 6.2 Algorithmic Bias and autonomy

Another issue with AI in music streaming is algorithmic bias. AI systems can sometimes reinforce narrow patterns of behavior, limiting the diversity of music users are exposed to. For instance, users may find themselves restricted to certain genres or artists based on past behavior, limiting their ability to explore new music. Balancing personalized recommendations with greater musical diversity is a challenge that platforms must address to avoid user fatigue.

Music recommendation systems play a significant role in influencing user experiences on music streaming platforms and have a strong impact on which artists are seen and those that fail because of the lack of visibility (Hesmondhalgh et al., 2023). Popular playlists on music streaming platforms are said to comprise mostly of artists from well-known recording companies further advancing their success, versus those artists from independent recording labels which also can lead to an algorithmic bias and their failure (Antal et al., 2021). There are increasing concerns in the music industry about bias algorithmic recommendations, where some stakeholders mention that it is in fact perpetuating inequality on the music streaming platforms (Wong, 2024).

Users have autonomy to choose or search for an artist on music streaming platforms, and recommendation engines offer suggestions based on historical behaviour, ultimately, the user has autonomy. Shank et al. (2023) research reveals how both music artists and music listeners tend to respond negatively to music produced by AI, showing negative perceptions and low likelihood of purchasing AI generated music. Bonicalzi et al. (2023) highlight that recommendation systems do assist users to navigate complex digital landscapes and platforms which can be overwhelming, they also have the power to influence users, reshape their identity and also pose as a threat of manipulation and deception.

Its imperative for music streaming platforms to aim continuously at creating an ethical ecosystem that benefits all stakeholders. This can be achieved by searching for ways to improve and solve algorithmic biases and ethical issues on recommendation systems by focusing on creating transparency, accountability and fairness systems. Bias mitigation techniques that deploy re-ranking models and combative debiasing can be integrated which aid in reducing recommendation systemic biases on DMSP (Torkashvand et al., 2023). Regular bias audits, where DMSP conduct regular periodic audits to review, detect and mitigate algorithmic biases to promote equal opportunity and ensure exposure is afforded to all artists across genres on the platform (Dinnissen and Bauer, 2023). Complying with General Data Protection Regulations, Spotify displays ethical disclosures to users, openly disclosing how and why data is collected, processed and implemented to influence recommendation systems on the platform (Soptify.com, 2021). Establishing trust in AI models is important (Ganatra et al., 2024), as seen with explainable AI which enhances transparency by explaining to users why certain recommendations appear.

Lakshan’s (2024) study on AI’s role on online music applications demonstrates how AI is reconfiguring the functionalities of music distribution, consumption and production through the effective use of natural language processing, machine learning and deep learning which assists in the advancement of personalized music recommendations, advance voice recognition and automated music generation. Prior studies have focused on mathematical strategies to manage algorithmic biases highlighting the pre - processing, in-processing and post-processing phases, however these computational procedures are not sufficient in addressing algorithmic biases as they do not capture the organizational, social and behavioral algorithmic biases aspect (Kordzadeh and Ghasemaghaei, 2022).

## 7 Conclusion and future trends

AI has fundamentally reshaped the digital music streaming industry, particularly in enhancing user experiences and driving subscription growth. Through AI-driven automation, platforms can deliver highly personalized music recommendations, optimize pricing strategies, and streamline customer service, all of which play pivotal roles in influencing consumer subscription responses. Personalization, powered by recommendation algorithms, keeps users engaged and fosters loyalty, which is crucial for retaining paying subscribers. Dynamic pricing models and targeted marketing campaigns further enhance user acquisition and conversion from free-tier to premium subscriptions.

Despite its many advantages, AI in digital music streaming also presents significant challenges. Privacy concerns, stemming from the vast amount of personal data collected, and the potential for algorithmic bias, which can limit users’ exposure to diverse music, are issues that platforms must address. As AI continues to evolve, music streaming platforms will need to balance the drive for hyper-personalization with ethical considerations around data security and fairness in recommendation systems.

**Figure 1:**
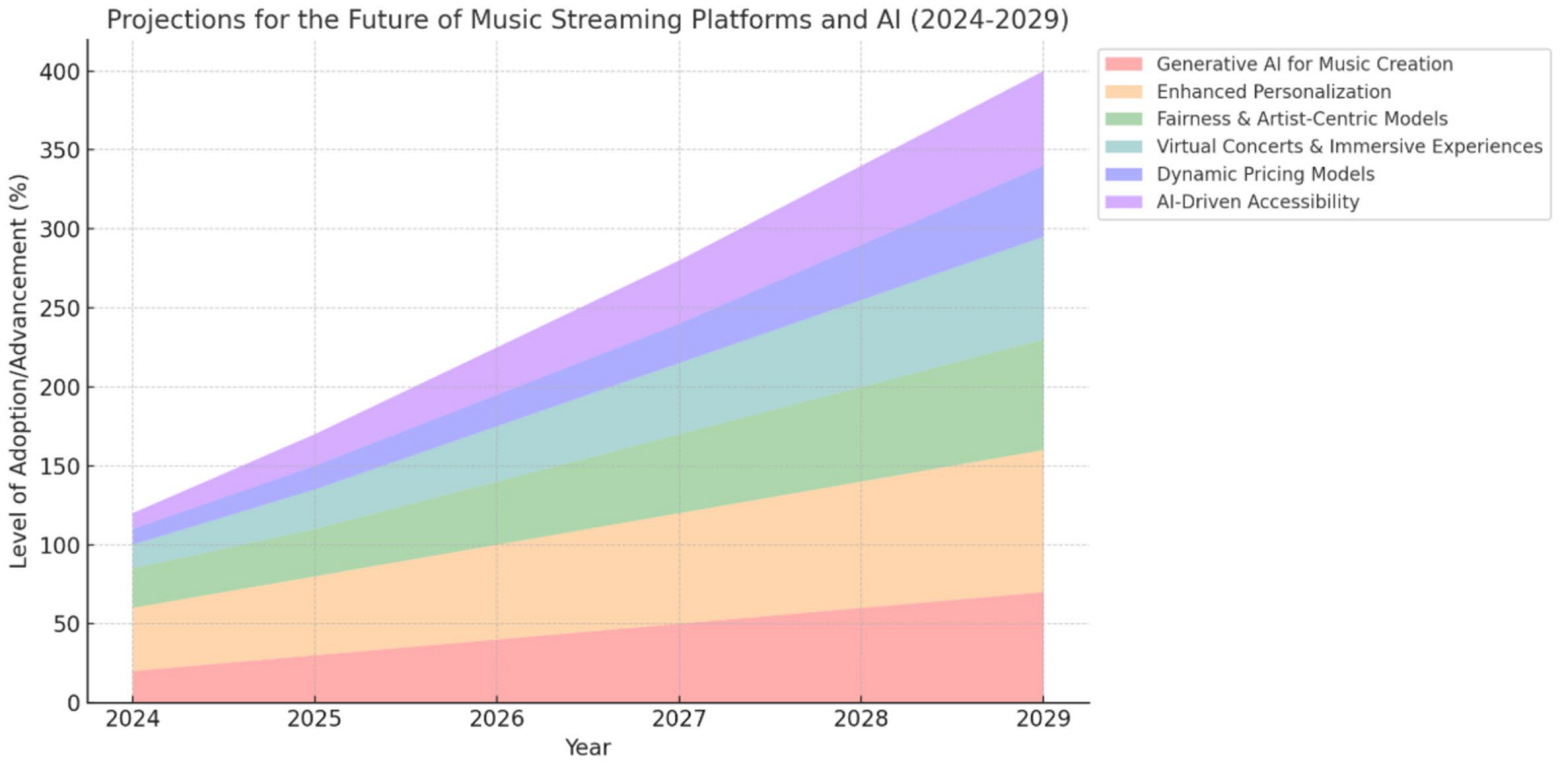
Projections for the future of digital music streaming platforms (DMSP) and the evolving role of AI (2024–2029). Data synthesized from Ganatra et al. (2024), Dinnissen and Bauer (2023) and Stål (2021).

Future trends and projections on the music streaming industry are key in strategically guiding DMSPs to ensure business continuity, sustainability and agility in adjusting and adapting to the advancement of AI while remaining relevant to the audience. As illustrated in Figure 1 the adoption of AI innovations on DMSP is projected to significantly grow across various domains. *Generative AI for music creation* will gradually increase as more DMSPs explore AI driven production and collaboration. *Enhanced Personalization* will gradually grow in sophistication as AI constantly refines user experience. The music streaming industry will experience a rising focus on *Fairness and Artist Centric Models* that promote fair royalty distribution and exposure for all artists on DMSPs. Future extension will include immersive music experiences through augmented reality (AR) and virtual reality (VR) integration. There will be an expanding adoption on immersive and interactive AI engagement for users, through *Virtual Concerts and Immersive Experience*s facilitated by AR and VR. The role of AI in digital music streaming will likely expand, with emerging technologies such as augmented reality and virtual reality creating collective metaverses offering new opportunities for immersive music experiences. A gradual adoption of *Dynamic Pricing Models* will be steadily adopted by more e-commerce and music streaming platforms focusing on real time pricing strategies utilising AI. Through *AI Driven Accessibility*, the music streaming industry will see more growth in tools and techniques deployed to promote music inclusivity across diverse users on DMSPs.

Looking ahead, efforts to create ethical AI frameworks will likely become more prominent to address concerns around privacy and algorithmic bias. The industry must focus on creating transparent, user-friendly policies around data usage and develop algorithms that promote diversity and inclusivity in music discovery. In doing so, platforms can continue to leverage AI’s potential while fostering ethical practices and maintaining consumer trust.
